# Metagenomics Reveals a Novel Virophage Population in a Tibetan Mountain Lake

**DOI:** 10.1264/jsme2.ME16003

**Published:** 2016-05-03

**Authors:** Seungdae Oh, Dongwan Yoo, Wen-Tso Liu

**Affiliations:** 1 School of Civil and Environmental Engineerin, Nanyang Technological UniversitySingaporeSingapore; 2 Department of Civil and Environmental Engineering, University of IllinoisUrbana-Champaign, Urbana, ILUSA; 3 Department of Pathobiology, University of IllinoisUrbana-Champaign, Urbana, ILUSA

**Keywords:** virophage, metagenomics, microbial ecology, Tibetan lake

## Abstract

Virophages are parasites of giant viruses that infect eukaryotic organisms and may affect the ecology of inland water ecosystems. Despite the potential ecological impact, limited information is available on the distribution, diversity, and hosts of virophages in ecosystems. Metagenomics revealed that virophages were widely distributed in inland waters with various environmental characteristics including salinity and nutrient availability. A novel virophage population was overrepresented in a planktonic microbial community of the Tibetan mountain lake, Lake Qinghai. Our study identified coccolithophores and coccolithovirus-like phycodnaviruses in the same community, which may serve as eukaryotic and viral hosts of the virophage population, respectively.

Virophages are small (<100 nm), icosahedral, double-stranded DNA viruses that replicate using the replication machinery of giant viruses (7, 14, 19). Virophages parasitize giant viruses, which is reflected in the formation of the abnormal particles of giant viruses and the increased survival rates of eukaryotic hosts when co-cultured (9, 14). Virophages have the ability to regulate members of *Mimivirus*, including *Acanthamoeba polyphaga mimivirus*, *Moumouvirus*, and *Megavirus chilensis*, which infect *A. polyphaga* (10). *Acanthamoeba* are commonly found in soil, fresh water, and seawater and are predators of various bacteria. Virophages control the populations of phototrophic algae by attenuating the infectivity of phycodnaviruses, which, in turn, may affect algal blooms (29). Thus, elucidating the distribution and host-virus interactions of virophages has important implications for nutrient cycling and food webs in natural aquatic ecosystems.

Only a few studies are available on virophage isolates and their host-virus interactions. Sputnik, the first virophage identified, was discovered with *A. polyphaga mimivirus*, which was isolated from a water sample taken from a cooling tower in France (14). Sputnik and its closest relatives (Sputnik2 and Suptnik3, the genome sequences of which share >99% nucleotide identity with the Sputnik genome) have a broad range of viral hosts among *Mimiviridae* that infect *Amoeba* (6, 10, 15). Zamilon is closely related to Sputnik (76% genome sequence identity) and has the ability to multiply in members of *Mimiviridae*. Zamilon and the giant virus Mont1 were co-isolated in a soil sample from Tunisia (11). Mavirus was isolated with the *Cafeteria roenbergensis* virus isolated from coastal waters in the USA (9). The Mavirus infection led to a decrease in the infectivity of the giant virus to the eukaryotic host, a marine phagotrophic flagellate.

Other virophages such as those found in Organic Lake (OLV), Yellowstone Lake (YSLV1 through YSLV4), and Ace Lake (ALM) were investigated by analyzing their genomes recovered from metagenomic datasets (29, 30). These studies provided insights into the viral and eukaryotic hosts of virophages and their co-evolution (*e.g.*, horizontal gene transfer among virophages, giant viruses, and eukaryotic hosts). Previous metagenomic studies documented the occurrence of virophages in diverse environments (fresh water, ocean, and soil) (5, 30) and the phylogenetic diversity of virophages in marine environments and Antarctic lakes (29). However, it has not yet been determined whether virophages are widely distributed in inland water environments, and, if so, what types of virophages are present. Therefore, addressing these issues is important in order to infer the ecological impact of virophages in inland water environments, given that characterized virophages are known to interact with different viral and eukaryotic hosts.

The metagenomic datasets of microbial communities sampled from the surface waters of ten lakes (Lanier, Mendota, Spark, Trout, Damariscotta, Vattern, Ekoln, Erken, Qinghai, and Yellowstone) and a river (Amazon) were collected (Table S1). Two Antarctic lakes (Ace and Organic) and a freshwater lagoon (Albufera) derived from seawater were also included. Major capsid protein (MCP) sequences are often used to classify viruses (3) and virophages (5, 24). Nine MCP sequences from all of the available virophage genomes were aligned using ClustalW (26). The sequence alignment identified a conserved region (amino acid positions 345–404; based on Sputnik) in the MCP sequences (Fig. S1). The metagenomic reads of the 13 datasets were searched against the conserved region using BLASTx (2) with cut-offs of >30% amino acid identity and >55 match length. The analysis revealed 134 virophage-like metagenomic reads originating from the majority (*n*=8) of the metagenomic datasets, indicating the widespread occurrence of virophages in inland waters with environmental gradients of salinity (freshwater to hypersaline) and nutrient availability (oligotrophic to eutrophic) (Table S1). The structure-based phylogenetic diversity of the virophages was investigated using the 134 virophage-like sequences (Fig. 1). While 92 sequences were closely related to those of the six previously characterized virophages (OLV, YSLV1 through 4, and ALM), the remaining 42 sequences corresponded to three distinct lineages (Lineages I, II, and III). Most of the newly identified virophage-like sequences (Lineages I and II) were more closely related (38 to 62% amino acid identity) to phycodnavirus-associated virophages (YSLVs and OLV) than Sputnik, Zamilon, and Mavirus.

Since many (*n*=26) of the virophage-like sequences of Lineage I identified in Qinghai were identical (100% identity) (Fig. 1), we attempted to reconstruct the genome sequence of the virophage population, designated as the Qinghai Lake virophage (QLV). The Qinghai metagenomic dataset was trimmed and assembled, as described previously (18). A search of all assembled contig sequences against the nine MCP sequences using BLASTx identified one MCP-encoding contig (contig_0049). Qinghai metagenomic reads were recruited to all assembled contigs using BLASTn with cut-offs of >95% nucleotide identity and >50% query length coverage, as described previously (16, 17). The read recruitment analysis identified metagenomic reads that simultaneously mapped on two distinct contigs. This approach was able to identify three additional contigs that were consecutively connected from contig_0049, based on the mapping patterns of the metagenomic reads. The metagenomic reads and four contigs were combined and re-assembled together using Newbler 2.8, which generated a single contig. The metagenomic reads were successively overlapped between the end and beginning of the single contig (Fig. S2), indicating that the single contig represents a circular genome, as with other virophages reported previously. Metagenomic read recruitment showed 9 to 100× coverage (56× on average) at 95–100% identities over the entire contig region. More than 93% of the recruited reads showed 98±2% identity, which was higher than the 90–95% average identities frequently used for phage species demarcation (1). These results suggest that the reconstructed QLV genome represents that of a species-like population, which has an intrapopulation genetic variation of approximately 2% (Fig. S2).

The length and G+C content of the QLV genome were 23,379 bp and 33.2% (Fig. 2), respectively. These genomic features were similar to those of other virophages reported previously as genome sizes of 17 to 28 kbp and G+C contents of 26 to 38% (9, 11, 14, 29, 30). The QLV genome encoded 25 open reading frames (ORFs) (Fig. 2 and Table S2). A gene content analysis identified genes commonly conserved between QLV and other virophage genomes: FtsK-HerA family ATPase (QLV1), cysteine protease (QLV6), MCP (QLV18), minor capsid protein (QLV19), and DNA helicase/primase/polymerase (QLV23) genes. The gene products of the core genes are known to carry out essential functions such as DNA replication and packaging in the virophage life cycle (11, 30). Phylogenetic trees were built using the full-length MCP, FtsK-HerA family ATPase, and cysteine protease genes (Fig. S3). The phylogenetic relationships observed based on the three different protein sequences were congruent with those shown in Fig. 1. These results confirmed the higher evolutionary relatedness of QLV to OLV-like virophages (OLV and YSLVs) than Sputnik and Mavirus, which corroborated the conserved region of the MCP sequences used in Fig. 1 serving as a robust genetic marker for a phylogenetic analysis.

QLV shared 7 (41% of the average amino acid identity), 8 (39%), 9 (40%), and 11 (46%) gene homologues with YSLV3, OLV, YSLV1, and YSLV4, respectively. QLV showed <35% average amino acid identity and <6 gene homologues with Sputnik, Zamilon, Mavirus, and ALM. QLV encoded 11 QLV-specific genes that were not found in other virophages when using cut-offs of 30% amino acid identity and 50% query length coverage (Fig. 2). While many of the predicted functions of the QLV-specific genes were hypothetical, QLV2 and QLV19 encoded a glycoprotein repeat domain-containing protein and RecB-family recombinase, respectively (Table S2). RecB is a subunit of the RecBCD enzyme that salvages double strand breaks in DNA through recombinational DNA repair (8); however, its exact role in virophages has not yet been investigated. Glycoproteins participate in the formation of the extracellular envelope, adhesion processes, and protein-protein interactions between viruses and their hosts (14, 29). A search of the QLV2 amino acid sequence against the non-redundant protein database (nr) showed the best hits (>48% amino acid identity) to phycodnaviruses (*Paramecium bursaria* and *Acanthocystis turfacea* Chlorella virus), which are known to infect unicellular green algae. Gene homologues conserved between virophages and their giant viruses were subjected to genetic exchange through the virophage-giant virus interaction (14, 29). Overall, the evolutionary relatedness of QLV to OLV-like virophages (Fig. 1 and S2) and the gene homology (QLV2) between QLV and phycodnaviruses (Table S2) collectively suggested that QLV presumably prey on phycodnaviruses.

The occurrence of QLV in the planktonic microbial community of Lake Qinghai may be interpreted by considering the filter size used for sampling and the size of virophages. Microbial cells smaller than 5 μm (in length) and larger than 0.22 μm were collected from a surface water sample taken from Lake Qinghai in a previous study (18). Since virophages are less than 0.1 μm in diameter, they may be underrepresented among the microbial cells collected using the above sample preparation. However, the QLV genome was overrepresented in the metagenomic dataset in terms of genome coverage (56×) relative to that of the other contigs assembled (<30×). Thus, it was speculated that QLV were sampled with their viral and/or eukaryotic hosts, in addition to those attached to and collected with suspended solids.

The Qinghai metagenomic reads were searched against the small subunit ribosomal RNA (SSU rRNA) gene (V9 hypervariable region) database (13) using BLASTn with cut-offs of >70% nucleotide identity and >90% target length coverage. More than 99% of the total SSU rRNA gene sequences retrieved were bacterial or archaeal, whereas four chloroplast 16S rRNA and two 18S rRNA gene sequences were identified (Fig. S4). The chloroplast 16S rRNA gene sequences were closely related (>98% identity) to coccolithophores (more specifically, *Isochrysis* spp. and *Emiliania huxleyi*). Coccolithophores are unicellular phytoplanktons that play a key role in nutrient cycling and food webs in water environments (21, 28). While *E. huxleyi* form extensive blooms from tropical to subpolar oceans, they are often found in oligotrophic environments. Some mechanisms (efficient cellular nitrogen utilization and ATP synthesis in nitrogen-limiting conditions) confer a fitness advantage to *E. huxleyi* in oligotrophic environments (22). Phytoplanktons have the ability to use alkaline phosphatase, which hydrolyzes extracellular inorganic phosphate, and this facilitates cellular uptake under phosphorous-limited conditions. The alkaline phosphatase of *E. huxleyi* exhibits maximum activity at approximately pH 9 (28). The surface waters (depth of 0.5 meters) of Lake Qinghai are oligotrophic (<1 mg L^−1^ of total nitrogen and <0.02 mg L^−1^ of total phosphorus) and alkaline (pH 9.3) (27). While *E. huxleyi* are commonly found in marine environments, they may have also been favored in Lake Qinghai due to, at least in part, the oligotrophic and alkaline conditions. Furthermore, we performed a metagenomic survey to ascertain the presence of coccolithophore-infecting viruses within the same microbial community. The alignment of MCP sequences from phycodnavirus genomes using ClustalW identified a conserved region (amino acid positions 433–492; based on Organic Lake phycodnavirus 1) in the MCP sequences. A search of the Qinghai metagenomic reads against the conserved MCP region using BLASTx with cut-offs of >30% identity and >90% target length coverage identified three MCP-encoding metagenomic reads. The three MCP sequences showed higher amino acid identities (31–40%) to *E. huxleyi* viruses than other phycodnaviruses (Fig. S5). These results suggest that phycodnavirus populations in the same microbial community were evolutionarily more closely related to *E. huxleyi* viruses, the parasites of the *E. huxleyi* observed in Fig. S4.

A PCR and metagenomic approach recently revealed a virophage population (DSLV1) in the surface waters of Lake Dishui in East China (12). Since DSLV1 showed genomic relatedness to YSLV3, it was associated with algae and algae-infecting large dsDNA viruses. QLV showed 35–51% amino acid identities on five gene homologues with DSLV1, suggesting marked genomic divergence between two virophage populations. The PCR amplification of DNA samples using MCP gene-specific primers revealed the occurrence of the DSLV1 population in Lake Dishui over a one-year period (12). Although PCR amplification is useful for identifying virophages closely related to the reference virophage strains used for primer design, it may not be useful for successfully detecting environmental virophages with marked genomic divergence (Fig. 1), as revealed in this study.

The development of a marker gene with high resolution within a specific viral group is essential for examining its diversity, distribution, and relative abundance in the environment, while viruses share no universal marker genes (*e.g.*, 16S rRNA genes in prokaryotes). A previous study searched metagenomic sequences using a cut-off of <10^−5^ E-value against “virophage-specific marker genes,” defined as those that had no hits to the nr database (30). In contrast, we selected a MCP gene as a genetic marker because it was one of the five core genes conserved among all characterized virophages that occurred in a single copy in a virophage genome and contained a 60-amino-acid-long region with the longest consecutive segment. We then searched raw metagenomic reads against the MCP alignment using cut-offs of >30% amino acid identity and >55 match length. Although our marker gene survey used a target region (60 amino acids), the amino acid length and cut-offs used were similar to the threshold (29–31% identities in 55–60 amino acids) for inferring structural homology between two proteins (23). In contrast to the 10^−3^–10^−6^ E-value cut-offs used in previous studies (5, 30), the 30% amino acid identity criterion was employed in the present study because it is considered to be more stringent and E-values change depending on the size of the database used (20). The 134 metagenomic reads (Fig. 1) retrieved in the present study showed best hits to the nine virophage MCP genes in the nr database, strongly suggesting that the 134 reads retrieved were of a virophage origin.

It is important to note that the occurrence and diversity of virophages (Fig. 1) in inland waters revealed in this study are underestimated because many of the sample preparations (Table S1) for metagenome sequencing were conducted using filter sizes larger than the typical size of virophages. Nevertheless, our metagenomic study detected a large number of virophage sequences in metagenomic datasets from many terrestrial aquatic environments using the robust genetic marker developed in the present study. Notably, we uncovered a novel virophage population particularly overrepresented in the planktonic microbial community of Lake Qinghai (Fig. 1 and 2). Distinctive from previous studies, our bioinformatic results detected eukaryotic and phycodnaviral populations as well as virophages in the same microbial community, which further implies that QLV are associated with coccolithophores and coccolithovirus-like phycodnaviruses. Therefore, we encourage future experiments determining the infectivity and host specificity of the novel virophage, which will contribute to more accurate assessments of the ecological consequences of the virophage population in the Tibetan mountain lake ecosystem.

## Nucleotide sequence accession numbers

The genome sequence of QLV was deposited in GenBank under the accession number KJ854379.1

## Supplementary Material



## Figures and Tables

**Fig. 1:**
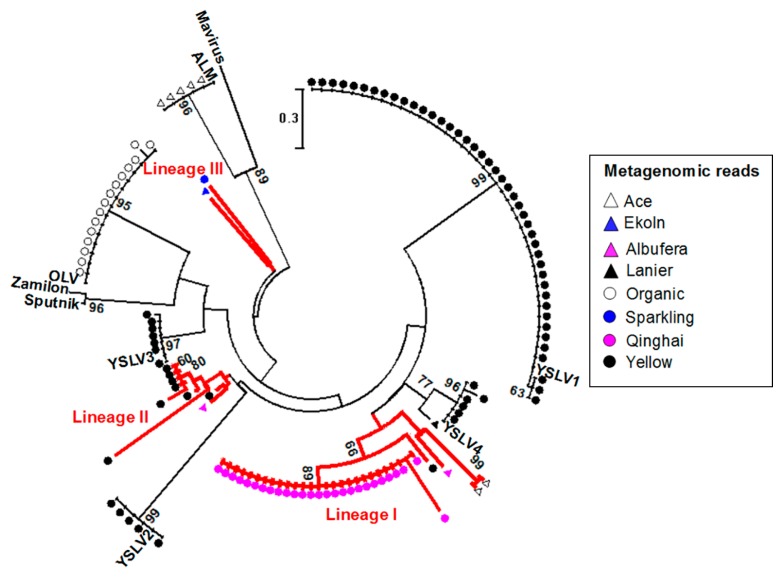
Occurrence and diversity of virophage populations in diverse terrestrial aquatic environments. The phylogenetic tree was built based on the maximum likelihood method with the Jones-Taylor-Thornton model using MEGA 6.0 (25). Bootstrap support values (higher than 50) from 100 replicates are shown on the nodes of the tree. The three lineages (Lineages I, II, and III) that were not closely related to the previously characterized virophages were highlighted.

**Fig. 2:**
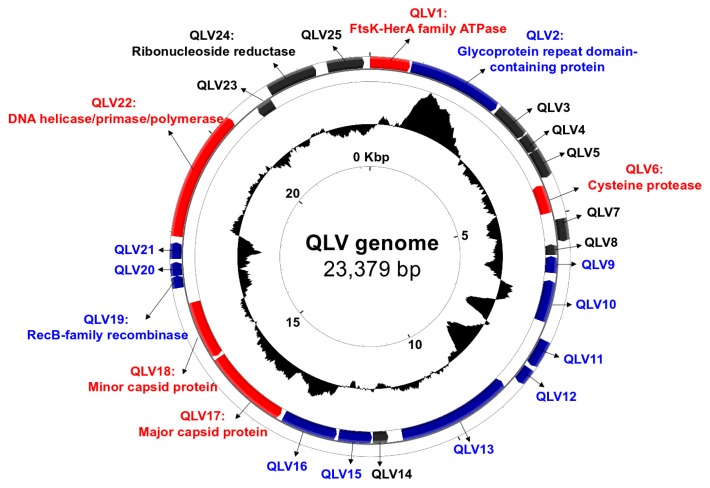
Circular map of the Qinghai Lake virophage (QLV) genome. Inwards: ORFs of the two DNA strands (red, blue, and black representing core, specific, and others, respectively) and G+C content. Protein-coding genes on the genome were predicted using GeneMark.hmm with the heuristic model (4). The protein-coding genes were functionally annotated by searching the amino acid sequences against the non-redundant protein database (nr) and metagenomic protein (env_nr) database, respectively, using BLASTp with >30% amino acid identity and >50% query length coverage.
