# Supplementary material for: Metagenomics Reveals a Novel Virophage Population in a Tibetan Mountain Lake
Source: Microbes Environ. 2016 May 3;31(2):173–7. doi: 10.1264/jsme2.ME16003 (PMC4912154; doi:10.1264/jsme2.ME16003)
Supplement: Supplementary file 1 [file 31_173_s1.pdf]

SUPPLEMENTARY MATERIAL

Metagenomics reveals a novel virophage population in a Tibetan mountain lake

Seungdae Oh, Dongwan Yoo, and Wen-Tso Liu

SUPPLEMENTARY TABLES

Table S1. Metagenomic datasets used in this study

ID	Sample source	Country	Sampling date	Size fraction (µm)	Sequencing technology[c]	Characteristics	Accession no.	Reference
Ekoln[a]	Lake Ekoln	Sweden	July/2007	0.2–100	454	Freshwater, eutrophic	ERX331315	(1)
Lanier[a]	Lake Lanier	USA	Aug/2009	0.22–1.6	454	Freshwater, mesotrophic	SRX025316	(4)
Sparkling[a]	Sparkling Lake	USA	May/2009	> 0.2	454	Freshwater, oligotrophic	ERX331320	(1)
Yellowstone[b]	Yellowstone Lake	USA	NA	NA	454	Freshwater, eutrophic	CAM_BM_001137<br>CAM_BM_001138<br>CAM_BM_001140<br>CAM_BM_001142<br>CAM_BM_001144<br>CAM_BM_001146<br>CAM_BM_001149	(7)
Qinghai[a]	Lake Qinghai	China	Sep/2007	0.22–5	454	Polysaline, oligotrophic	SRR1290860	(5)
Albufera[b]	Albufera Lagoon	Spain	May/2010	> 0.1<br>> 0.3<br>> 0.8	454	Freshwater, eutrophic	CAM_SMPL_003228<br>CAM_SMPL_003229<br>CAM_SMPL_003230	(2)
Organic[b]	Organic Lake	Antarctica	Dec/2006	> 0.1	Sanger	Hypersaline, eutrophic	4443685.3	(8)

Ace[b]	Ace Lake	Antarctica	Dec/2006	> 0.1	Sanger	Varied salinities (freshwater to hypersaline along with the depth)	4443683.3<br>4443680.3<br>4443679.3<br>4443681.3<br>4443682.3	(8)
Mendota[a]	Lake Mendota	USA	May/2009<br>Aug/2009	> 0.2	454	Freshwater, eutrophic	ERX331322 (May)<br>ERX331321 (Aug)	(1)
Damariscotta[a]	Lake Damariscotta	USA	May/2009<br>Aug/2009	> 0.2	454	Freshwater, mesotrophic	ERX331317 (May)<br>ERX331318 (Aug)	(1)
Trout[a]	Trout Bog Lake	USA	May/2007	> 0.2	454	Freshwater, dysotrophic	ERX331319	(1)
Vattern[a]	Lake Vättern	Sweden	Jul/2007	0.2–100	454	Freshwater, oligotrophic	ERX331316	(1)
Erken[a]	Lake Erken	Sweden	Jun/2007	0.2–100	454	Freshwater, mesotrophic	ERX331314	(1)
Amazon[a]	Amazon River	Brazil	Sep/2008	0.22-5	454	Freshwater	SRX037805	(3)

Metagenomic datasets were obtained from the GenBank[a] (http://ftp.ncbi.nih.gov/) or MG-RAST[b] (metagenomics.anl.gov/) database. [c]454 and Sanger represent the Roche 454 pyrosequencing and Sanger sequencing technology, respectively, used for metagenome sequencing. These datasets were chosen since the sample sources represented a range of terrestrial aquatic environments at the time of analysis and the datasets had long (average read length > 300 bp) raw sequence reads (i.e., sequenced by the Roche 454 pyrosequencing or Sanger sequencing technology). * NA - not available.

Table S2. Gene products of ORFs predicted in the QLV genome

Gene ID	Length[a]	Gene product	Coverage[c] (%)	Identity[d] (%)	Locus tag
QLV1	257	Ftsk-HerA family ATPase	99	58	YSLV4_01
QLV2	607	Glycoprotein repeat domain-containing protein	63	48	ATCVOR07043_945L
QLV3	232	Hypothetical	65	40	YSLV1_07
QLV4	115	Hypothetical	97	50	YSLV4_14
QLV5	186	Hypothetical	100	47	YSLV4_14
QLV6	198	Cysteine protease	94	44	YSLV1_23
QLV7	147	Hypothetical	91	44	YSLV4_17
QLV8	73	Hypothetical[b]	51	35	GOS_8066434
QLV9	114	ORFan[e]	NA	NA	NA
QLV10	284	ORFan[e]	NA	NA	NA
QLV11	181	ORFan[e]	NA	NA	NA
QLV12	112	Hypothetical[b]	72	32	GOS_1486477
QLV13	785	ORFan[e]	NA	NA	NA
QLV14	106	Hypothetical	99	52	YSLV4_26
QLV15	228	ORFan[e]	NA	NA	NA
QLV16	384	ORFan[e]	NA	NA	NA
QLV17	578	Major capsid protein	100	45	YSLV4_22
QLV18	400	Minor capsid protein	97	43	YSLV4_21
QLV19	79	Recombination protein	55	32	Hac_0287
QLV20	87	Two component transcriptional regulator protein[b]	47	39	B2A_02913
QLV21	104	Hypothetical	91	32	WP_009216564
QLV22	840	DNA helicase/primase/polymerase	53	30	PGVV_00004
QLV23	117	Hypothetical	97	57	YSLV3_06
QLV24	327	Ribonucleoside reductase	93	66	DICPUDRAFT_56591

QLV25	237	Hypothetical	98	34	YSLV4_34

25 ORFs with > 60 amino acid sequence length[a] were predicted in the QLV genome. The gene product was inferred based on its best BLASTp match analysis against the non-redundant (nr) protein database or environmental (env_nr)[b] protein database (in case no hits against the nr database), using a cutoff of > 50% query length coverage[c] and > 30% amino acid identity[d] . Similar results (gene prediction and annotation) were observed using the MetaVir web server (6). ORFan[e] genes are referred to here as those that were predicted as ORFs but were found to have no gene homologues in the above protein databases.

SUPPLEMENTARY FIGURES

```
YSLV4    QNYTSSFLAINGISVNFNNASGLLSTATQQDLYNISYANGSSQTFHEFRGFADVNNNNSG
YSLV1    MSAAPSFLAINNISVNLNNTSGLLASCSSSDLYRISVANHSNQTFSAWSGVFLADAGITD
Sputnik  ITTPDVFLQINNLNLTWNNQQGILSGASSQNLYDFSVQNGYNKTWSEFNGVTQQFNGVSG
Zamilon  ITTPDVFLQINSLNLTWNNQQGVLSGASAQNLYDFSVQNGYNKTWTEFNGLTQQLSGVSG
YSLV3    NFDSDSFLPITGITINFNNKAGLLSGATQWDLWRMSVESGSNQTWAEFQGFAPLGSQTAY
ALM      VYQANAFFPIKNISINTDNRNNLLENLDSYQLYMLSSKNGLNKRPSTFLGSAADPTSADE
Mavirus  KFAPDQLAGIIGLEIKVDSDVGIFRELEQQQLYELSSSNGYNKRFSCFSGALANGLTVAD
OLV      YWSNNLSYPISQVNITFNNKAGLLSEMDAYSLYQMSRRNGSQQTWNEFRGVVRSGNGGKF
YSLV2    LGISDFRFPVKNVNITWNTQSGILSNAQPQELYLMSKRGGLAQDFLSWRGKATADGDGTA
                  :  : :. :.  .::      .*: :*  .   :    : *
```

Figure S1. Alignment of virophage MCP sequences. Asterisks, colons, and periods indicate positions with identical residues, conserved substitutions of residues, and semiconserved substitutions of residues, respectively. This region (positions 345-404 in amino acids; based on Sputnik) contained the longest consecutive segment alignable.

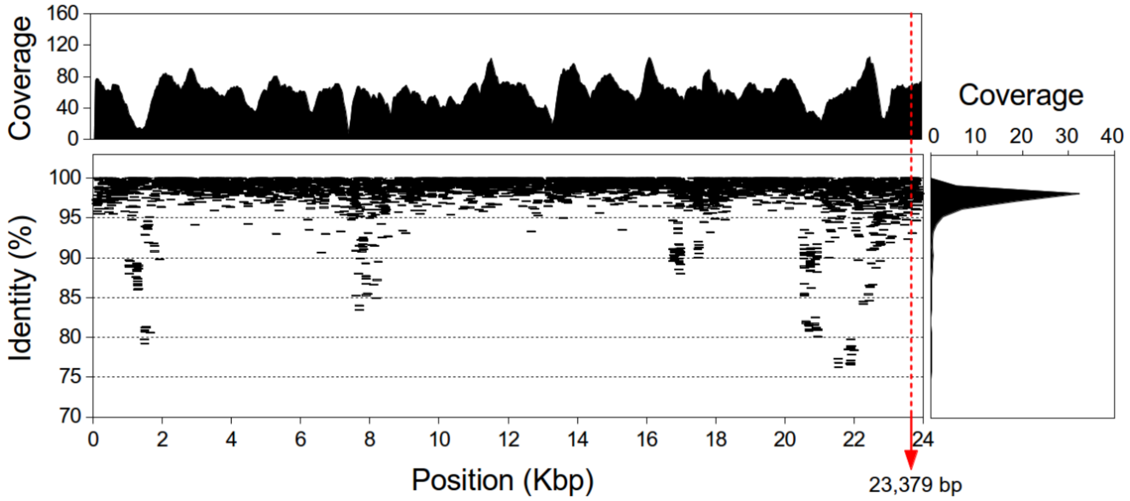

Figure S2. Metagenomic read recruitment plot. The graph shows the nucleotide identity of each metagenomic read against the QLV genome with > 70% identity and > 100 match length. The identity was plotted on the positions of each read on the genome. The beginning (position 1 to 300) of the genome was artificially concatenated at the end of the genome (23,379) as indicated with a red arrow, which resulted in the 23,679 bp sequence. The graph on the top panel displays the metagenomic read coverage (9 to 100×) over the whole genomic region without any gaps. The graph on the right panel shows the metagenomic read coverage at a given unit of nucleotide identity. Note the highest coverage at 98% identity and the total coverage of 56× (i.e., area) at 95-100% identities.

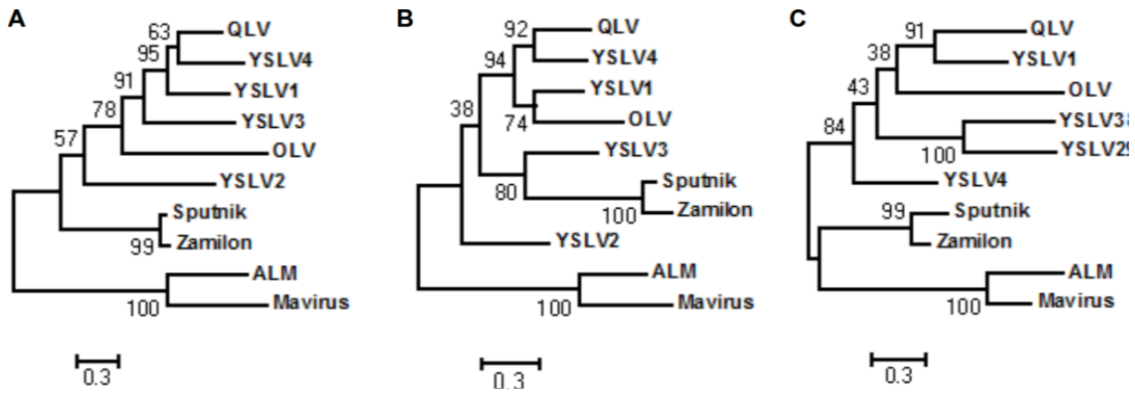

Figure S3. Phylogenetic tree of ten virophages based on the full amino acid sequences of MCP (A), FtsK-HerA family ATPase (B), and cysteine protease (C). The phylogenetic trees were built using MEGA 6.0 as described in Fig. 1.

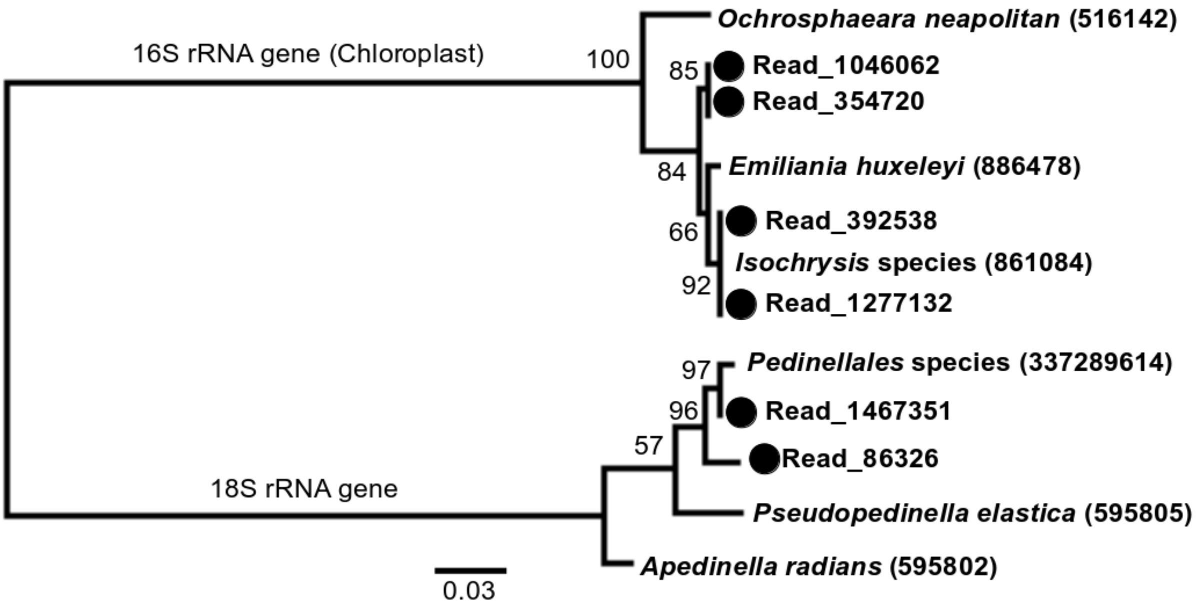

Figure S4. Phylogenetic tree of small subunit ribosomal RNA (SSU rRNA) gene sequences recovered from Qinghai. The phylogenetic tree was built based on the maximum likelihood method with the Tamura-Nei model using MEGA 6.0. The black circles represent the metagenomic reads recovered. Representative SSU ribosomal gene sequences from the nucleotide collection database (nr/nt) in GenBank were included for comparison; the GI numbers are provided in parentheses.

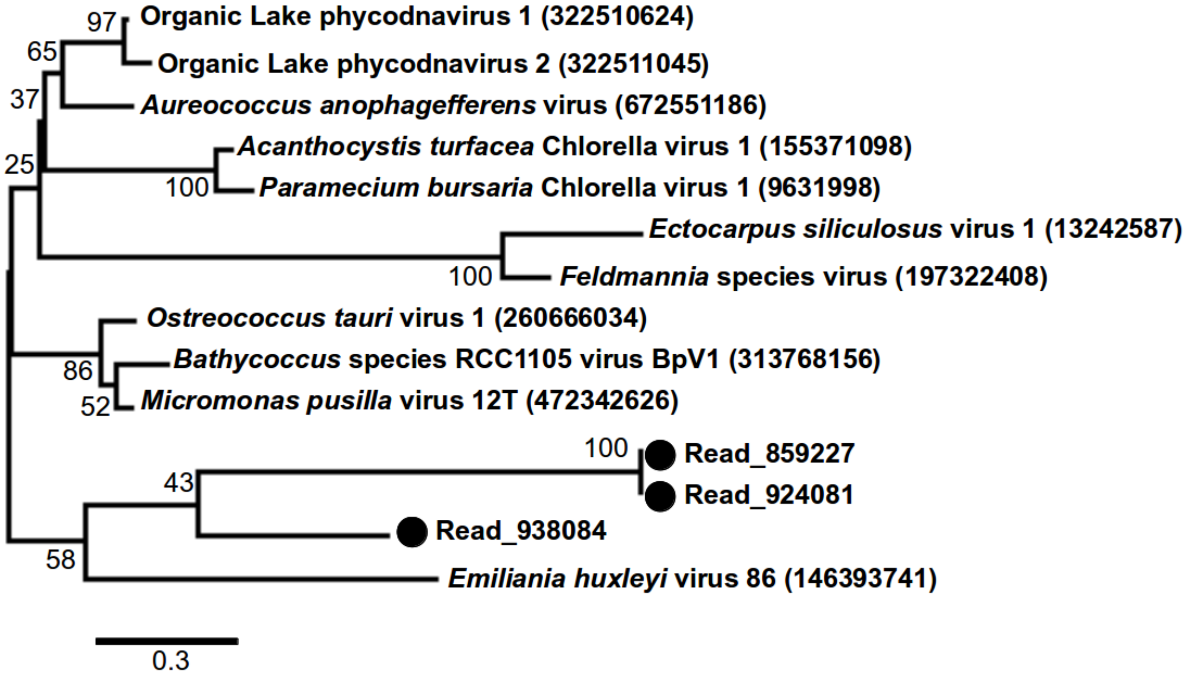

Figure S5. Phylogenetic tree of phycodnavirus major capsid protein (MCP) sequences identified in Qinghai. The phylogenetic tree was constructed as described in Fig. 1. The black circles represent the translated amino acid sequences of the metagenomic reads recovered from Qinghai. Representative MCP sequences (positions 433-492; based on Organic Lake phycodnavirus 1) of all sequenced phycodnavirus genomes from GenBank (ftp://ftp.ncbi.nih.gov) were included for comparison. The GI numbers are provided in parentheses.

References

1. Eiler, A., Zaremba-Niedzwiedzka, K., Garcia, M.M., McMahon, K.D., Stepanauskas, R., Andersson, S.G., and Bertilsson, S. 2014. Productivity and salinity structuring of the microplankton revealed by comparative freshwater metagenomics. Environ. Microbiol. 16: 2682-2698.

2. Ghai, R., Hernandez, C.M., Picazo, et al. 2012. Metagenomes of mediterranean coastal lagoons. Sci. Rep. 2:490.

3. Ghai, R., Rodriguez-Valera, F., McMahon, K.D., Toyama, D., Rinke, R., Cristina Souza de Oliveira, T., Wagner Garcia, J., Pellon de Miranda, F., and Henrique-Silva, F. 2011. Metagenomics of the water column in the pristine upper course of the amazon river. PLoS ONE. 6:e23785.

4. Oh, S., Caro-Quintero, A., Tsementzi, D., DeLeon-Rodriguez, N., Luo, C., Poretsky, R., and Konstantinidis, K.T. 2011. Metagenomic insights into the evolution, function, and complexity of the planktonic microbial community of Lake Lanier, a temperate freshwater ecosystem. Appl. Environ. Microbiol. 77: 6000–6011.

5. Oh, S., Zhang, R., Wu, Q., and Liu, W. 2014. Draft genome sequence of a novel SAR11 clade species abundant in a tibetan lake. Genome Announc. 2:10–11.

6. Roux, S., J.Tournayre, A.Mahul, D.Debroas, and F.Enault. 2014. Metavir 2: new tools for viral metagenome comparison and assembled virome analysis. BMC Bioinformatics. 15:76.

7. Rusch, D.B., Halpern, A.L., Sutton, G., et al. 2007. The sorcerer II global ocean sampling expedition: northwest atlantic through eastern tropical pacific. PLoS Biol. 5:0398–0431.

8. Yau, S., Lauro, F.M., Williams, T.J., Demaere, M.Z., Brown, M.V., Rich, J., Gibson, J.A., and Cavicchioli, R. 2013. Metagenomic insights into strategies of carbon conservation and unusual sulfur biogeochemistry in a hypersaline antarctic lake. ISME J. 7:1944–1961.
